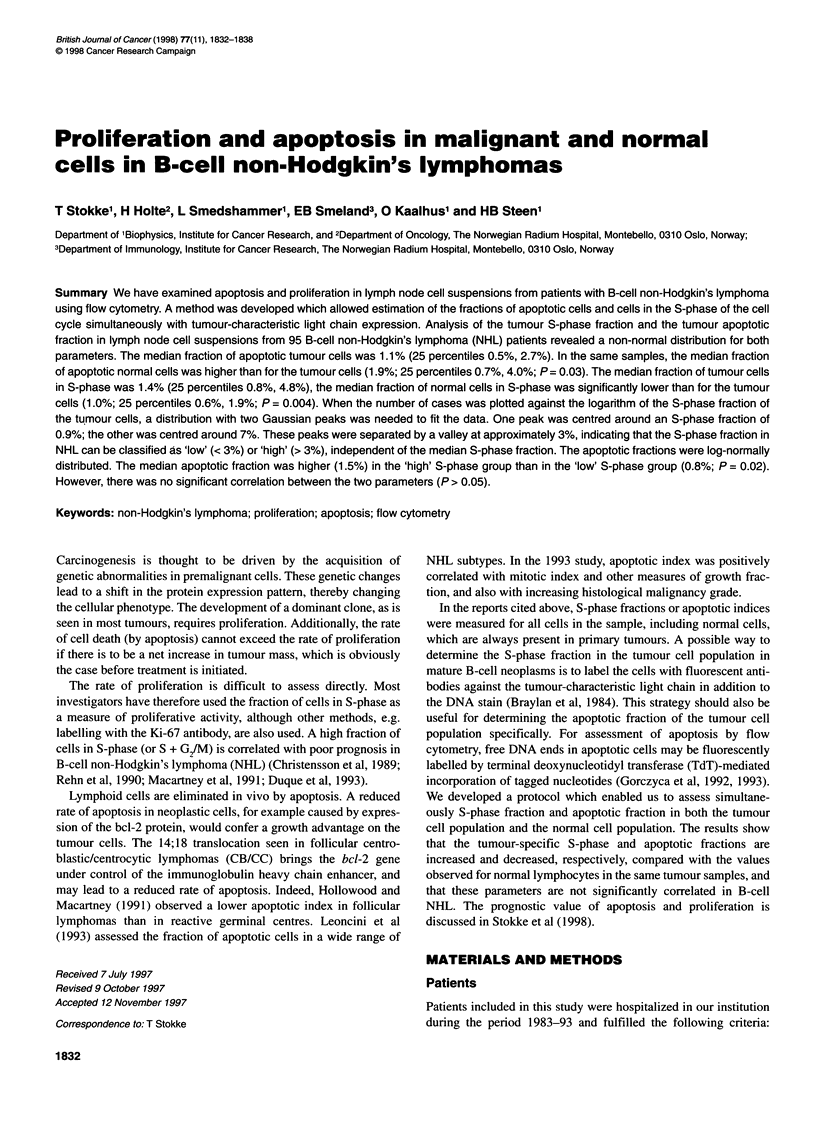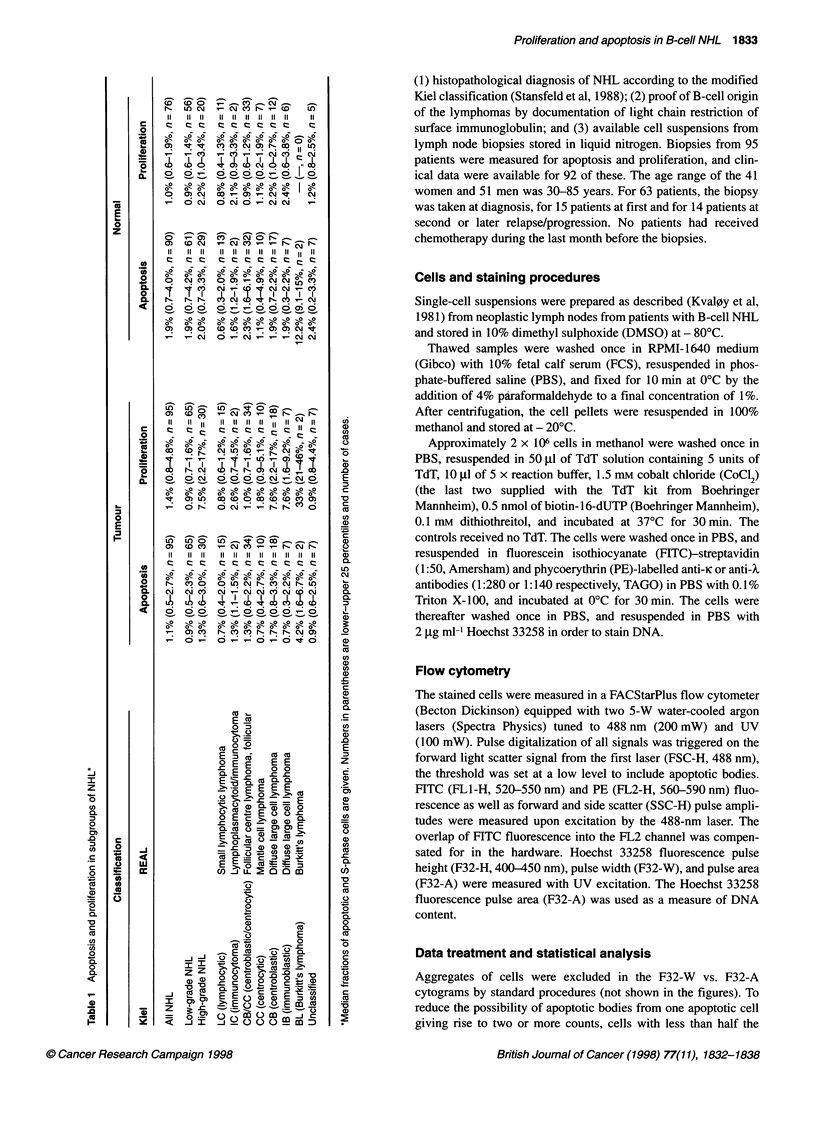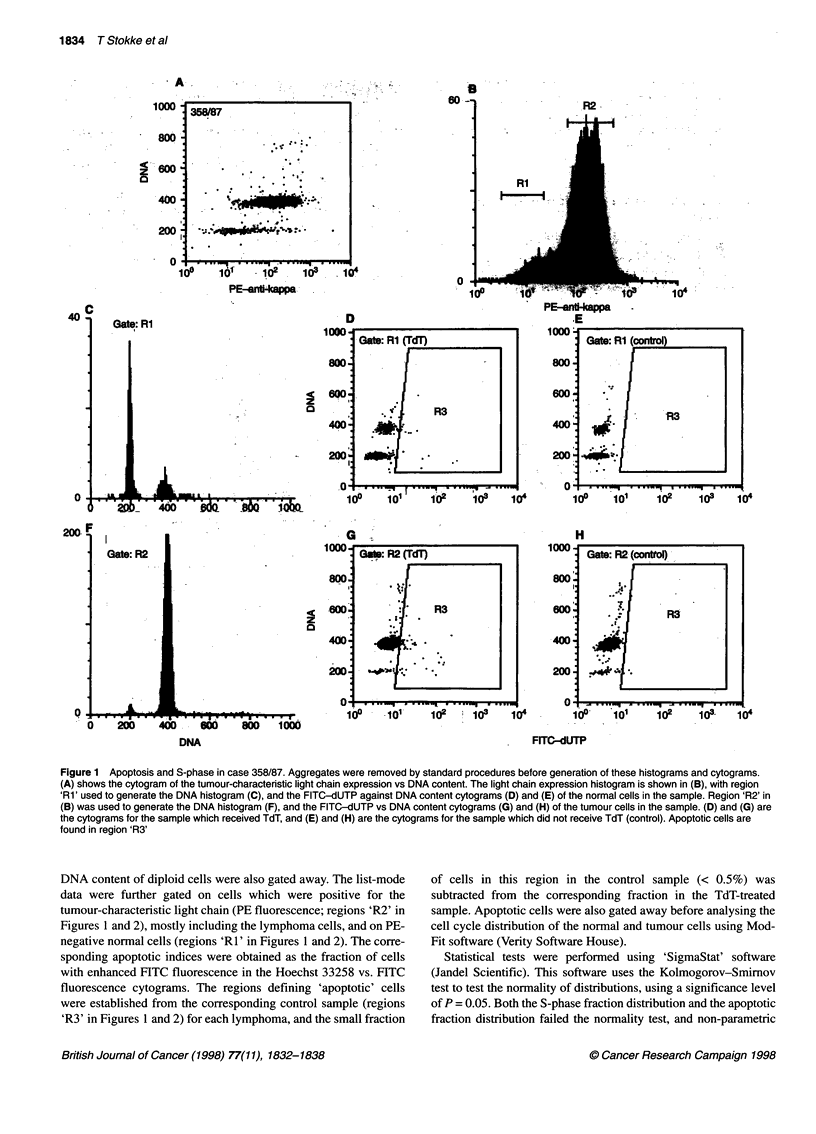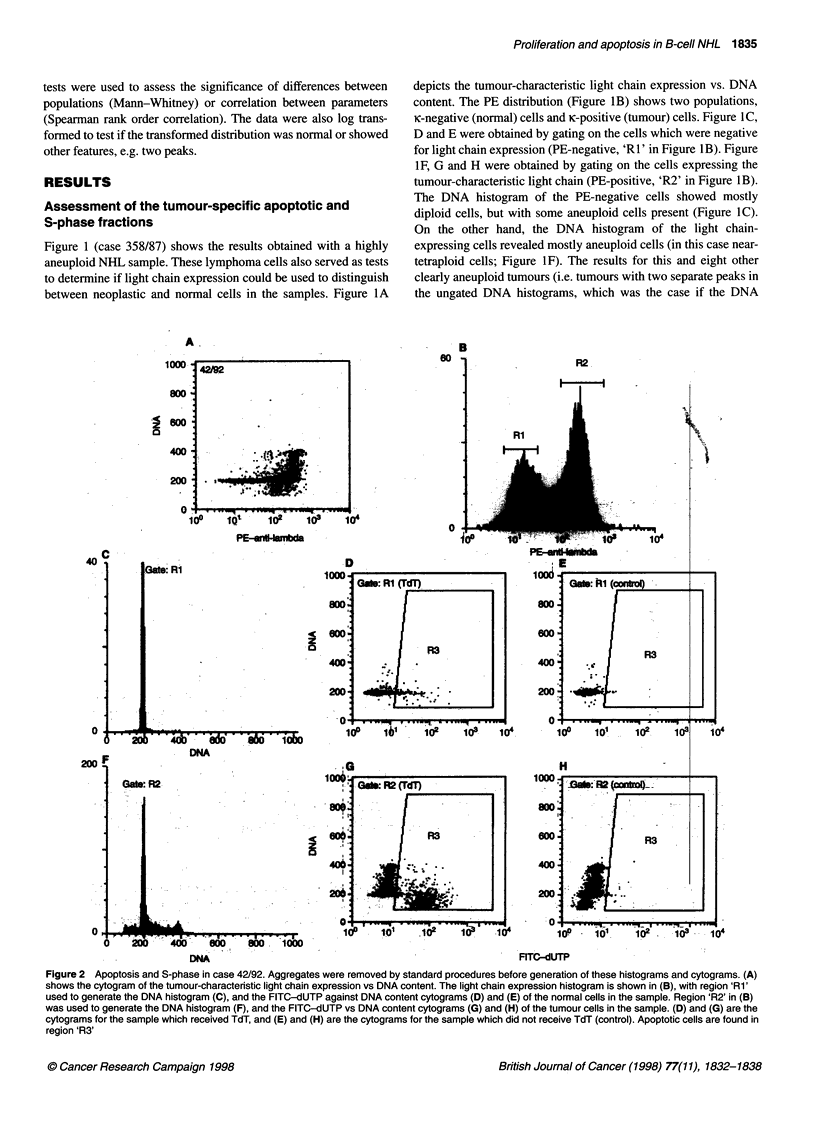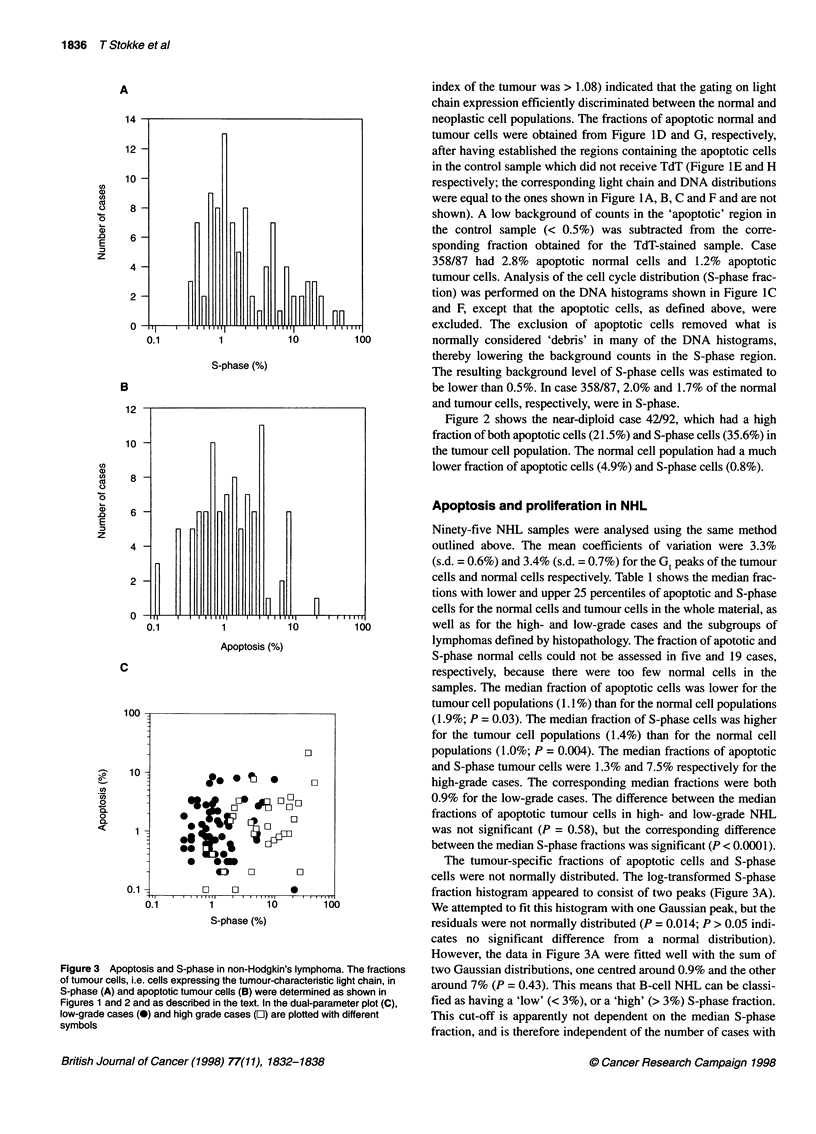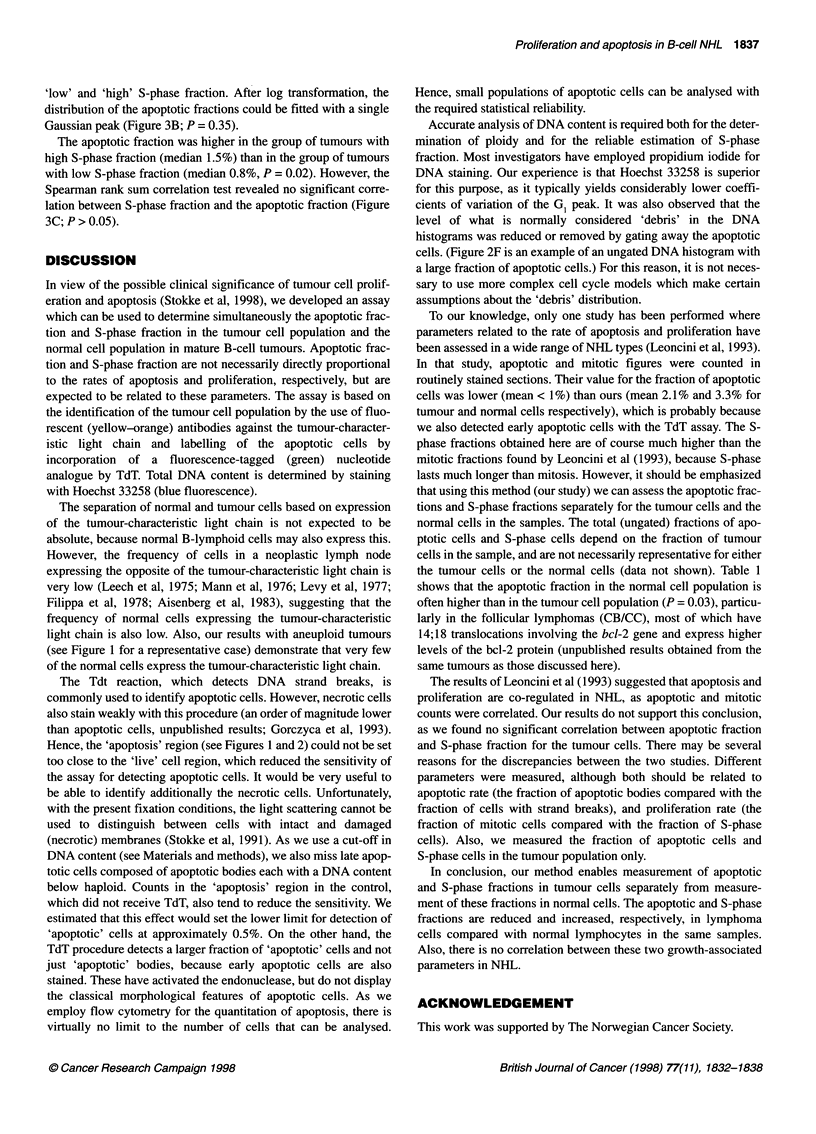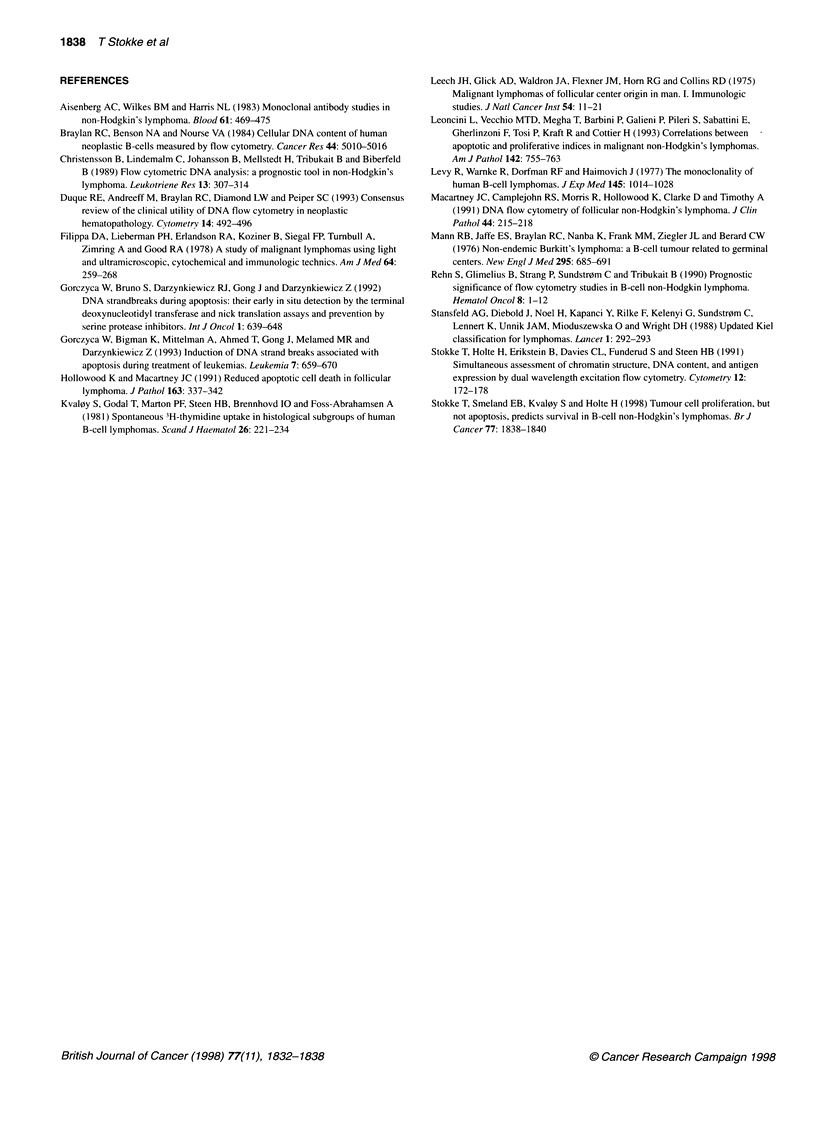# Proliferation and apoptosis in malignant and normal cells in B-cell non-Hodgkin's lymphomas.

**DOI:** 10.1038/bjc.1998.304

**Published:** 1998-06

**Authors:** T. Stokke, H. Holte, L. Smedshammer, E. B. Smeland, O. Kaalhus, H. B. Steen

**Affiliations:** Department of Biophysics, Institute for Cancer Research, The Norwegian Radium Hospital, Montebello.

## Abstract

We have examined apoptosis and proliferation in lymph node cell suspensions from patients with B-cell non-Hodgkin's lymphoma using flow cytometry. A method was developed which allowed estimation of the fractions of apoptotic cells and cells in the S-phase of the cell cycle simultaneously with tumour-characteristic light chain expression. Analysis of the tumour S-phase fraction and the tumour apoptotic fraction in lymph node cell suspensions from 95 B-cell non-Hodgkin's lymphoma (NHL) patients revealed a non-normal distribution for both parameters. The median fraction of apoptotic tumour cells was 1.1% (25 percentiles 0.5%, 2.7%). In the same samples, the median fraction of apoptotic normal cells was higher than for the tumour cells (1.9%; 25 percentiles 0.7%, 4.0%; P = 0.03). The median fraction of tumour cells in S-phase was 1.4% (25 percentiles 0.8%, 4.8%), the median fraction of normal cells in S-phase was significantly lower than for the tumour cells (1.0%; 25 percentiles 0.6%, 1.9%; P = 0.004). When the number of cases was plotted against the logarithm of the S-phase fraction of the tumour cells, a distribution with two Gaussian peaks was needed to fit the data. One peak was centred around an S-phase fraction of 0.9%; the other was centred around 7%. These peaks were separated by a valley at approximately 3%, indicating that the S-phase fraction in NHL can be classified as 'low' (< 3%) or 'high' (> 3%), independent of the median S-phase fraction. The apoptotic fractions were log-normally distributed. The median apoptotic fraction was higher (1.5%) in the 'high' S-phase group than in the 'low' S-phase group (0.8%; P = 0.02). However, there was no significant correlation between the two parameters (P > 0.05).


British Journal of Cancer (1998) 77(11), 1832–1838
© 1998 Cancer Research Campaign

### Proliferation and apoptosis in malignant and normal cells in B-cell non-Hodgkin's lymphomas

**T Stokke[1], H Holte[2], L Smedshammer[1], EB Smeland[3], O Kaalhus[1] and HB Steen[1]**

Department of [1]Biophysics, Institute for Cancer Research, and [2]Department of Oncology, The Norwegian Radium Hospital, Montebello, 0310 Oslo, Norway; [3]Department of Immunology, Institute for Cancer Research, The Norwegian Radium Hospital, Montebello, 0310 Oslo, Norway

**Summary** We have examined apoptosis and proliferation in lymph node cell suspensions from patients with B-cell non-Hodgkin's lymphoma using flow cytometry. A method was developed which allowed estimation of the fractions of apoptotic cells and cells in the S-phase of the cell cycle simultaneously with tumour-characteristic light chain expression. Analysis of the tumour S-phase fraction and the tumour apoptotic fraction in lymph node cell suspensions from 95 B-cell non-Hodgkin's lymphoma (NHL) patients revealed a non-normal distribution for both parameters. The median fraction of apoptotic tumour cells was 1.1% (25 percentiles 0.5%, 2.7%). In the same samples, the median fraction of apoptotic normal cells was higher than for the tumour cells (1.9%; 25 percentiles 0.7%, 4.0%; $P = 0.03$). The median fraction of tumour cells in S-phase was 1.4% (25 percentiles 0.8%, 4.8%), the median fraction of normal cells in S-phase was significantly lower than for the tumour cells (1.0%; 25 percentiles 0.6%, 1.9%; $P = 0.004$). When the number of cases was plotted against the logarithm of the S-phase fraction of the tumour cells, a distribution with two Gaussian peaks was needed to fit the data. One peak was centred around an S-phase fraction of 0.9%; the other was centred around 7%. These peaks were separated by a valley at approximately 3%, indicating that the S-phase fraction in NHL can be classified as 'low' (< 3%) or 'high' (> 3%), independent of the median S-phase fraction. The apoptotic fractions were log-normally distributed. The median apoptotic fraction was higher (1.5%) in the 'high' S-phase group than in the 'low' S-phase group (0.8%; $P = 0.02$). However, there was no significant correlation between the two parameters ($P > 0.05$).

**Keywords:** non-Hodgkin's lymphoma; proliferation; apoptosis; flow cytometry

Carcinogenesis is thought to be driven by the acquisition of genetic abnormalities in premalignant cells. These genetic changes lead to a shift in the protein expression pattern, thereby changing the cellular phenotype. The development of a dominant clone, as is seen in most tumours, requires proliferation. Additionally, the rate of cell death (by apoptosis) cannot exceed the rate of proliferation if there is to be a net increase in tumour mass, which is obviously the case before treatment is initiated.

The rate of proliferation is difficult to assess directly. Most investigators have therefore used the fraction of cells in S-phase as a measure of proliferative activity, although other methods, e.g. labelling with the Ki-67 antibody, are also used. A high fraction of cells in S-phase (or S + $G_2$/M) is correlated with poor prognosis in B-cell non-Hodgkin's lymphoma (NHL) (Christensson et al, 1989; Rehn et al, 1990; Macartney et al, 1991; Duque et al, 1993).

Lymphoid cells are eliminated in vivo by apoptosis. A reduced rate of apoptosis in neoplastic cells, for example caused by expression of the bcl-2 protein, would confer a growth advantage on the tumour cells. The 14;18 translocation seen in follicular centroblastic/centrocytic lymphomas (CB/CC) brings the *bcl*-2 gene under control of the immunoglobulin heavy chain enhancer, and may lead to a reduced rate of apoptosis. Indeed, Hollowood and Macartney (1991) observed a lower apoptotic index in follicular lymphomas than in reactive germinal centres. Leoncini et al (1993) assessed the fraction of apoptotic cells in a wide range of NHL subtypes. In the 1993 study, apoptotic index was positively correlated with mitotic index and other measures of growth fraction, and also with increasing histological malignancy grade.

In the reports cited above, S-phase fractions or apoptotic indices were measured for all cells in the sample, including normal cells, which are always present in primary tumours. A possible way to determine the S-phase fraction in the tumour cell population in mature B-cell neoplasms is to label the cells with fluorescent antibodies against the tumour-characteristic light chain in addition to the DNA stain (Braylan et al, 1984). This strategy should also be useful for determining the apoptotic fraction of the tumour cell population specifically. For assessment of apoptosis by flow cytometry, free DNA ends in apoptotic cells may be fluorescently labelled by terminal deoxynucleotidyl transferase (TdT)-mediated incorporation of tagged nucleotides (Gorczyca et al, 1992, 1993). We developed a protocol which enabled us to assess simultaneously S-phase fraction and apoptotic fraction in both the tumour cell population and the normal cell population. The results show that the tumour-specific S-phase and apoptotic fractions are increased and decreased, respectively, compared with the values observed for normal lymphocytes in the same tumour samples, and that these parameters are not significantly correlated in B-cell NHL. The prognostic value of apoptosis and proliferation is discussed in Stokke et al (1998).

*Received 7 July 1997*
*Revised 9 October 1997*
*Accepted 12 November 1997*

*Correspondence to:* T Stokke

### MATERIALS AND METHODS

### Patients

Patients included in this study were hospitalized in our institution during the period 1983–93 and fulfilled the following criteria:

**Table 1** Apoptosis and proliferation in subgroups of NHL*

| Classification | | Tumour | | Normal | |
|---|---|---|---|---|---|
| Kiel | REAL | Apoptosis | Proliferation | Apoptosis | Proliferation |
| All NHL | | 1.1% (0.5–2.7%, *n* = 95) | 1.4% (0.8–4.8%, *n* = 95) | 1.9% (0.7–4.0%, *n* = 90) | 1.0% (0.6–1.9%, *n* = 76) |
| Low-grade NHL | | 0.9% (0.5–2.3%, *n* = 65) | 0.9% (0.7–1.6%, *n* = 65) | 1.9% (0.7–4.2%, *n* = 61) | 0.9% (0.6–1.4%, *n* = 56) |
| High-grade NHL | | 1.3% (0.6–3.0%, *n* = 30) | 7.5% (2.2–17%, *n* = 30) | 2.0% (0.7–3.3%, *n* = 29) | 2.2% (1.0–3.4%, *n* = 20) |
| LC (lymphocytic) | Small lymphocytic lymphoma | 0.7% (0.4–2.0%, *n* = 15) | 0.8% (0.6–1.2%, *n* = 15) | 0.6% (0.3–2.0%, *n* = 13) | 0.8% (0.4–1.3%, *n* = 11) |
| IC (immunocytoma) | Lymphoplasmacytoid/immunocytoma | 1.3% (1.1–1.5%, *n* = 2) | 2.6% (0.7–4.5%, *n* = 2) | 1.6% (1.2–1.9%, *n* = 2) | 2.1% (0.9–3.3%, *n* = 2) |
| CB/CC (centroblastic/centrocytic) | Follicular centre lymphoma, follicular | 1.3% (0.6–2.2%, *n* = 34) | 1.0% (0.7–1.6%, *n* = 34) | 2.3% (1.6–6.1%, *n* = 32) | 0.9% (0.6–1.2%, *n* = 33) |
| CC (centrocytic) | Mantle cell lymphoma | 0.7% (0.4–2.7%, *n* = 10) | 1.8% (0.9–5.1%, *n* = 10) | 1.1% (0.4–4.9%, *n* = 10) | 1.1% (0.2–1.9%, *n* = 7) |
| CB (centroblastic) | Diffuse large cell lymphoma | 1.7% (0.8–3.3%, *n* = 18) | 7.6% (2.2–17%, *n* = 18) | 1.9% (0.7–2.2%, *n* = 17) | 2.2% (1.0–2.7%, *n* = 12) |
| IB (immunoblastic) | Diffuse large cell lymphoma | 0.7% (0.3–2.2%, *n* = 7) | 7.6% (1.6–9.2%, *n* = 7) | 1.9% (0.3–2.2%, *n* = 7) | 2.4% (0.6–3.8%, *n* = 6) |
| BL (Burkitt's lymphoma) | Burkitt's lymphoma | 4.2% (1.6–6.7%, *n* = 2) | 33% (21–46%, *n* = 2) | 12.2% (9.1–15%, *n* = 2) | — (—, *n* = 0) |
| Unclassified | | 0.9% (0.6–2.5%, *n* = 7) | 0.9% (0.8–4.4%, *n* = 7) | 2.4% (0.2–3.3%, *n* = 7) | 1.2% (0.8–2.5%, *n* = 5) |

*Median fractions of apoptotic and S-phase cells are given. Numbers in parentheses are lower–upper 25 percentiles and number of cases.

(1) histopathological diagnosis of NHL according to the modified Kiel classification (Stansfeld et al, 1988); (2) proof of B-cell origin of the lymphomas by documentation of light chain restriction of surface immunoglobulin; and (3) available cell suspensions from lymph node biopsies stored in liquid nitrogen. Biopsies from 95 patients were measured for apoptosis and proliferation, and clinical data were available for 92 of these. The age range of the 41 women and 51 men was 30–85 years. For 63 patients, the biopsy was taken at diagnosis, for 15 patients at first and for 14 patients at second or later relapse/progression. No patients had received chemotherapy during the last month before the biopsies.

### Cells and staining procedures

Single-cell suspensions were prepared as described (Kvaløy et al, 1981) from neoplastic lymph nodes from patients with B-cell NHL and stored in 10% dimethyl sulphoxide (DMSO) at – 80°C.

Thawed samples were washed once in RPMI-1640 medium (Gibco) with 10% fetal calf serum (FCS), resuspended in phosphate-buffered saline (PBS), and fixed for 10 min at 0°C by the addition of 4% paraformaldehyde to a final concentration of 1%. After centrifugation, the cell pellets were resuspended in 100% methanol and stored at – 20°C.

Approximately $2 \times 10^6$ cells in methanol were washed once in PBS, resuspended in 50 μl of TdT solution containing 5 units of TdT, 10 μl of 5 × reaction buffer, 1.5 mM cobalt chloride ($CoCl_2$) (the last two supplied with the TdT kit from Boehringer Mannheim), 0.5 nmol of biotin-16-dUTP (Boehringer Mannheim), 0.1 mM dithiothreitol, and incubated at 37°C for 30 min. The controls received no TdT. The cells were washed once in PBS, and resuspended in fluorescein isothiocyanate (FITC)–streptavidin (1:50, Amersham) and phycoerythrin (PE)-labelled anti-κ or anti-λ antibodies (1:280 or 1:140 respectively, TAGO) in PBS with 0.1% Triton X-100, and incubated at 0°C for 30 min. The cells were thereafter washed once in PBS, and resuspended in PBS with 2 μg $ml^{-1}$ Hoechst 33258 in order to stain DNA.

### Flow cytometry

The stained cells were measured in a FACStarPlus flow cytometer (Becton Dickinson) equipped with two 5-W water-cooled argon lasers (Spectra Physics) tuned to 488 nm (200 mW) and UV (100 mW). Pulse digitalization of all signals was triggered on the forward light scatter signal from the first laser (FSC-H, 488 nm), the threshold was set at a low level to include apoptotic bodies. FITC (FL1-H, 520–550 nm) and PE (FL2-H, 560–590 nm) fluorescence as well as forward and side scatter (SSC-H) pulse amplitudes were measured upon excitation by the 488-nm laser. The overlap of FITC fluorescence into the FL2 channel was compensated for in the hardware. Hoechst 33258 fluorescence pulse height (F32-H, 400–450 nm), pulse width (F32-W), and pulse area (F32-A) were measured with UV excitation. The Hoechst 33258 fluorescence pulse area (F32-A) was used as a measure of DNA content.

### Data treatment and statistical analysis

Aggregates of cells were excluded in the F32-W vs. F32-A cytograms by standard procedures (not shown in the figures). To reduce the possibility of apoptotic bodies from one apoptotic cell giving rise to two or more counts, cells with less than half the

© Cancer Research Campaign 1998

**Figure 1** Apoptosis and S-phase in case 358/87. Aggregates were removed by standard procedures before generation of these histograms and cytograms. (**A**) shows the cytogram of the tumour-characteristic light chain expression vs DNA content. The light chain expression histogram is shown in (**B**), with region 'R1' used to generate the DNA histogram (**C**), and the FITC–dUTP against DNA content cytograms (**D**) and (**E**) of the normal cells in the sample. Region 'R2' in (**B**) was used to generate the DNA histogram (**F**), and the FITC–dUTP vs DNA content cytograms (**G**) and (**H**) of the tumour cells in the sample. (**D**) and (**G**) are the cytograms for the sample which received TdT, and (**E**) and (**H**) are the cytograms for the sample which did not receive TdT (control). Apoptotic cells are found in region 'R3'

DNA content of diploid cells were also gated away. The list-mode data were further gated on cells which were positive for the tumour-characteristic light chain (PE fluorescence; regions 'R2' in Figures 1 and 2), mostly including the lymphoma cells, and on PE-negative normal cells (regions 'R1' in Figures 1 and 2). The corresponding apoptotic indices were obtained as the fraction of cells with enhanced FITC fluorescence in the Hoechst 33258 vs. FITC fluorescence cytograms. The regions defining 'apoptotic' cells were established from the corresponding control sample (regions 'R3' in Figures 1 and 2) for each lymphoma, and the small fraction of cells in this region in the control sample (< 0.5%) was subtracted from the corresponding fraction in the TdT-treated sample. Apoptotic cells were also gated away before analysing the cell cycle distribution of the normal and tumour cells using Mod-Fit software (Verity Software House).

Statistical tests were performed using 'SigmaStat' software (Jandel Scientific). This software uses the Kolmogorov–Smirnov test to test the normality of distributions, using a significance level of $P = 0.05$. Both the S-phase fraction distribution and the apoptotic fraction distribution failed the normality test, and non-parametric

 © Cancer Research Campaign 1998

tests were used to assess the significance of differences between populations (Mann–Whitney) or correlation between parameters (Spearman rank order correlation). The data were also log transformed to test if the transformed distribution was normal or showed other features, e.g. two peaks.

### RESULTS

### Assessment of the tumour-specific apoptotic and S-phase fractions

Figure 1 (case 358/87) shows the results obtained with a highly aneuploid NHL sample. These lymphoma cells also served as tests to determine if light chain expression could be used to distinguish between neoplastic and normal cells in the samples. Figure 1A depicts the tumour-characteristic light chain expression vs. DNA content. The PE distribution (Figure 1B) shows two populations, κ-negative (normal) cells and κ-positive (tumour) cells. Figure 1C, D and E were obtained by gating on the cells which were negative for light chain expression (PE-negative, 'R1' in Figure 1B). Figure 1F, G and H were obtained by gating on the cells expressing the tumour-characteristic light chain (PE-positive, 'R2' in Figure 1B). The DNA histogram of the PE-negative cells showed mostly diploid cells, but with some aneuploid cells present (Figure 1C). On the other hand, the DNA histogram of the light chain-expressing cells revealed mostly aneuploid cells (in this case near-tetraploid cells; Figure 1F). The results for this and eight other clearly aneuploid tumours (i.e. tumours with two separate peaks in the ungated DNA histograms, which was the case if the DNA

**Figure 2** Apoptosis and S-phase in case 42/92. Aggregates were removed by standard procedures before generation of these histograms and cytograms. (**A**) shows the cytogram of the tumour-characteristic light chain expression vs DNA content. The light chain expression histogram is shown in (**B**), with region 'R1' used to generate the DNA histogram (**C**), and the FITC–dUTP against DNA content cytograms (**D**) and (**E**) of the normal cells in the sample. Region 'R2' in (**B**) was used to generate the DNA histogram (**F**), and the FITC–dUTP vs DNA content cytograms (**G**) and (**H**) of the tumour cells in the sample. (**D**) and (**G**) are the cytograms for the sample which received TdT, and (**E**) and (**H**) are the cytograms for the sample which did not receive TdT (control). Apoptotic cells are found in region 'R3'

**Figure 3** Apoptosis and S-phase in non-Hodgkin's lymphoma. The fractions of tumour cells, i.e. cells expressing the tumour-characteristic light chain, in S-phase (**A**) and apoptotic tumour cells (**B**) were determined as shown in Figures 1 and 2 and as described in the text. In the dual-parameter plot (**C**), low-grade cases (●) and high grade cases (□) are plotted with different symbols index of the tumour was > 1.08) indicated that the gating on light chain expression efficiently discriminated between the normal and neoplastic cell populations. The fractions of apoptotic normal and tumour cells were obtained from Figure 1D and G, respectively, after having established the regions containing the apoptotic cells in the control sample which did not receive TdT (Figure 1E and H respectively; the corresponding light chain and DNA distributions were equal to the ones shown in Figure 1A, B, C and F and are not shown). A low background of counts in the 'apoptotic' region in the control sample (< 0.5%) was subtracted from the corresponding fraction obtained for the TdT-stained sample. Case 358/87 had 2.8% apoptotic normal cells and 1.2% apoptotic tumour cells. Analysis of the cell cycle distribution (S-phase fraction) was performed on the DNA histograms shown in Figure 1C and F, except that the apoptotic cells, as defined above, were excluded. The exclusion of apoptotic cells removed what is normally considered 'debris' in many of the DNA histograms, thereby lowering the background counts in the S-phase region. The resulting background level of S-phase cells was estimated to be lower than 0.5%. In case 358/87, 2.0% and 1.7% of the normal and tumour cells, respectively, were in S-phase.

Figure 2 shows the near-diploid case 42/92, which had a high fraction of both apoptotic cells (21.5%) and S-phase cells (35.6%) in the tumour cell population. The normal cell population had a much lower fraction of apoptotic cells (4.9%) and S-phase cells (0.8%).

### Apoptosis and proliferation in NHL

Ninety-five NHL samples were analysed using the same method outlined above. The mean coefficients of variation were 3.3% (s.d. = 0.6%) and 3.4% (s.d. = 0.7%) for the $G_1$ peaks of the tumour cells and normal cells respectively. Table 1 shows the median fractions with lower and upper 25 percentiles of apoptotic and S-phase cells for the normal cells and tumour cells in the whole material, as well as for the high- and low-grade cases and the subgroups of lymphomas defined by histopathology. The fraction of apototic and S-phase normal cells could not be assessed in five and 19 cases, respectively, because there were too few normal cells in the samples. The median fraction of apoptotic cells was lower for the tumour cell populations (1.1%) than for the normal cell populations (1.9%; $P = 0.03$). The median fraction of S-phase cells was higher for the tumour cell populations (1.4%) than for the normal cell populations (1.0%; $P = 0.004$). The median fractions of apoptotic and S-phase tumour cells were 1.3% and 7.5% respectively for the high-grade cases. The corresponding median fractions were both 0.9% for the low-grade cases. The difference between the median fractions of apoptotic tumour cells in high- and low-grade NHL was not significant ($P = 0.58$), but the corresponding difference between the median S-phase fractions was significant ($P < 0.0001$).

The tumour-specific fractions of apoptotic cells and S-phase cells were not normally distributed. The log-transformed S-phase fraction histogram appeared to consist of two peaks (Figure 3A). We attempted to fit this histogram with one Gaussian peak, but the residuals were not normally distributed ($P = 0.014$; $P > 0.05$ indicates no significant difference from a normal distribution). However, the data in Figure 3A were fitted well with the sum of two Gaussian distributions, one centred around 0.9% and the other around 7% ($P = 0.43$). This means that B-cell NHL can be classified as having a 'low' (< 3%), or a 'high' (> 3%) S-phase fraction. This cut-off is apparently not dependent on the median S-phase fraction, and is therefore independent of the number of cases with

© Cancer Research Campaign 1998

'low' and 'high' S-phase fraction. After log transformation, the distribution of the apoptotic fractions could be fitted with a single Gaussian peak (Figure 3B; $P = 0.35$).

The apoptotic fraction was higher in the group of tumours with high S-phase fraction (median 1.5%) than in the group of tumours with low S-phase fraction (median 0.8%, $P = 0.02$). However, the Spearman rank sum correlation test revealed no significant correlation between S-phase fraction and the apoptotic fraction (Figure 3C; $P > 0.05$).

### DISCUSSION

In view of the possible clinical significance of tumour cell proliferation and apoptosis (Stokke et al, 1998), we developed an assay which can be used to determine simultaneously the apoptotic fraction and S-phase fraction in the tumour cell population and the normal cell population in mature B-cell tumours. Apoptotic fraction and S-phase fraction are not necessarily directly proportional to the rates of apoptosis and proliferation, respectively, but are expected to be related to these parameters. The assay is based on the identification of the tumour cell population by the use of fluorescent (yellow–orange) antibodies against the tumour-characteristic light chain and labelling of the apoptotic cells by incorporation of a fluorescence-tagged (green) nucleotide analogue by TdT. Total DNA content is determined by staining with Hoechst 33258 (blue fluorescence).

The separation of normal and tumour cells based on expression of the tumour-characteristic light chain is not expected to be absolute, because normal B-lymphoid cells may also express this. However, the frequency of cells in a neoplastic lymph node expressing the opposite of the tumour-characteristic light chain is very low (Leech et al, 1975; Mann et al, 1976; Levy et al, 1977; Filippa et al, 1978; Aisenberg et al, 1983), suggesting that the frequency of normal cells expressing the tumour-characteristic light chain is also low. Also, our results with aneuploid tumours (see Figure 1 for a representative case) demonstrate that very few of the normal cells express the tumour-characteristic light chain.

The Tdt reaction, which detects DNA strand breaks, is commonly used to identify apoptotic cells. However, necrotic cells also stain weakly with this procedure (an order of magnitude lower than apoptotic cells, unpublished results; Gorczyca et al, 1993). Hence, the 'apoptosis' region (see Figures 1 and 2) could not be set too close to the 'live' cell region, which reduced the sensitivity of the assay for detecting apoptotic cells. It would be very useful to be able to identify additionally the necrotic cells. Unfortunately, with the present fixation conditions, the light scattering cannot be used to distinguish between cells with intact and damaged (necrotic) membranes (Stokke et al, 1991). As we use a cut-off in DNA content (see Materials and methods), we also miss late apoptotic cells composed of apoptotic bodies each with a DNA content below haploid. Counts in the 'apoptosis' region in the control, which did not receive TdT, also tend to reduce the sensitivity. We estimated that this effect would set the lower limit for detection of 'apoptotic' cells at approximately 0.5%. On the other hand, the TdT procedure detects a larger fraction of 'apoptotic' cells and not just 'apoptotic' bodies, because early apoptotic cells are also stained. These have activated the endonuclease, but do not display the classical morphological features of apoptotic cells. As we employ flow cytometry for the quantitation of apoptosis, there is virtually no limit to the number of cells that can be analysed. Hence, small populations of apoptotic cells can be analysed with the required statistical reliability.

Accurate analysis of DNA content is required both for the determination of ploidy and for the reliable estimation of S-phase fraction. Most investigators have employed propidium iodide for DNA staining. Our experience is that Hoechst 33258 is superior for this purpose, as it typically yields considerably lower coefficients of variation of the $G_1$ peak. It was also observed that the level of what is normally considered 'debris' in the DNA histograms was reduced or removed by gating away the apoptotic cells. (Figure 2F is an example of an ungated DNA histogram with a large fraction of apoptotic cells.) For this reason, it is not necessary to use more complex cell cycle models which make certain assumptions about the 'debris' distribution.

To our knowledge, only one study has been performed where parameters related to the rate of apoptosis and proliferation have been assessed in a wide range of NHL types (Leoncini et al, 1993). In that study, apoptotic and mitotic figures were counted in routinely stained sections. Their value for the fraction of apoptotic cells was lower (mean < 1%) than ours (mean 2.1% and 3.3% for tumour and normal cells respectively), which is probably because we also detected early apoptotic cells with the TdT assay. The S-phase fractions obtained here are of course much higher than the mitotic fractions found by Leoncini et al (1993), because S-phase lasts much longer than mitosis. However, it should be emphasized that using this method (our study) we can assess the apoptotic fractions and S-phase fractions separately for the tumour cells and the normal cells in the samples. The total (ungated) fractions of apoptotic cells and S-phase cells depend on the fraction of tumour cells in the sample, and are not necessarily representative for either the tumour cells or the normal cells (data not shown). Table 1 shows that the apoptotic fraction in the normal cell population is often higher than in the tumour cell population ($P = 0.03$), particularly in the follicular lymphomas (CB/CC), most of which have 14;18 translocations involving the *bcl*-2 gene and express higher levels of the bcl-2 protein (unpublished results obtained from the same tumours as those discussed here).

The results of Leoncini et al (1993) suggested that apoptosis and proliferation are co-regulated in NHL, as apoptotic and mitotic counts were correlated. Our results do not support this conclusion, as we found no significant correlation between apoptotic fraction and S-phase fraction for the tumour cells. There may be several reasons for the discrepancies between the two studies. Different parameters were measured, although both should be related to apoptotic rate (the fraction of apoptotic bodies compared with the fraction of cells with strand breaks), and proliferation rate (the fraction of mitotic cells compared with the fraction of S-phase cells). Also, we measured the fraction of apoptotic cells and S-phase cells in the tumour population only.

In conclusion, our method enables measurement of apoptotic and S-phase fractions in tumour cells separately from measurement of these fractions in normal cells. The apoptotic and S-phase fractions are reduced and increased, respectively, in lymphoma cells compared with normal lymphocytes in the same samples. Also, there is no correlation between these two growth-associated parameters in NHL.

### ACKNOWLEDGEMENT

This work was supported by The Norwegian Cancer Society.

© Cancer Research Campaign 1998

### REFERENCES

Aisenberg AC, Wilkes BM and Harris NL (1983) Monoclonal antibody studies in non-Hodgkin's lymphoma. *Blood* **61**: 469–475

Braylan RC, Benson NA and Nourse VA (1984) Cellular DNA content of human neoplastic B-cells measured by flow cytometry. *Cancer Res* **44**: 5010–5016

Christensson B, Lindemalm C, Johansson B, Mellstedt H, Tribukait B and Biberfeld B (1989) Flow cytometric DNA analysis: a prognostic tool in non-Hodgkin's lymphoma. *Leukotriene Res* **13**: 307–314

Duque RE, Andreeff M, Braylan RC, Diamond LW and Peiper SC (1993) Consensus review of the clinical utility of DNA flow cytometry in neoplastic hematopathology. *Cytometry* **14**: 492–496

Filippa DA, Lieberman PH, Erlandson RA, Koziner B, Siegal FP, Turnbull A, Zimring A and Good RA (1978) A study of malignant lymphomas using light and ultramicroscopic, cytochemical and immunologic technics. *Am J Med* **64**: 259–268

Gorczyca W, Bruno S, Darzynkiewicz RJ, Gong J and Darzynkiewicz Z (1992) DNA strandbreaks during apoptosis: their early in situ detection by the terminal deoxynucleotidyl transferase and nick translation assays and prevention by serine protease inhibitors. *Int J Oncol* **1**: 639–648

Gorczyca W, Bigman K, Mittelman A, Ahmed T, Gong J, Melamed MR and Darzynkiewicz Z (1993) Induction of DNA strand breaks associated with apoptosis during treatment of leukemias. *Leukemia* **7**: 659–670

Hollowood K and Macartney JC (1991) Reduced apoptotic cell death in follicular lymphoma. *J Pathol* **163**: 337–342

Kvaløy S, Godal T, Marton PF, Steen HB, Brennhovd IO and Foss-Abrahamsen A (1981) Spontaneous $^{3}$H-thymidine uptake in histological subgroups of human B-cell lymphomas. *Scand J Haematol* **26**: 221–234

Leech JH, Glick AD, Waldron JA, Flexner JM, Horn RG and Collins RD (1975) Malignant lymphomas of follicular center origin in man. I. Immunologic studies. *J Natl Cancer Inst* **54**: 11–21

Leoncini L, Vecchio MTD, Megha T, Barbini P, Galieni P, Pileri S, Sabattini E, Gherlinzoni F, Tosi P, Kraft R and Cottier H (1993) Correlations between apoptotic and proliferative indices in malignant non-Hodgkin's lymphomas. *Am J Pathol* **142**: 755–763

Levy R, Warnke R, Dorfman RF and Haimovich J (1977) The monoclonality of human B-cell lymphomas. *J Exp Med* **145**: 1014–1028

Macartney JC, Camplejohn RS, Morris R, Hollowood K, Clarke D and Timothy A (1991) DNA flow cytometry of follicular non-Hodgkin's lymphoma. *J Clin Pathol* **44**: 215–218

Mann RB, Jaffe ES, Braylan RC, Nanba K, Frank MM, Ziegler JL and Berard CW (1976) Non-endemic Burkitt's lymphoma: a B-cell tumour related to germinal centers. *New Engl J Med* **295**: 685–691

Rehn S, Glimelius B, Strang P, Sundstrøm C and Tribukait B (1990) Prognostic significance of flow cytometry studies in B-cell non-Hodgkin lymphoma. *Hematol Oncol* **8**: 1–12

Stansfeld AG, Diebold J, Noel H, Kapanci Y, Rilke F, Kelenyi G, Sundstrøm C, Lennert K, Unnik JAM, Mioduszewska O and Wright DH (1988) Updated Kiel classification for lymphomas. *Lancet* **1**: 292–293

Stokke T, Holte H, Erikstein B, Davies CL, Funderud S and Steen HB (1991) Simultaneous assessment of chromatin structure, DNA content, and antigen expression by dual wavelength excitation flow cytometry. *Cytometry* **12**: 172–178

Stokke T, Smeland EB, Kvaløy S and Holte H (1998) Tumour cell proliferation, but not apoptosis, predicts survival in B-cell non-Hodgkin's lymphomas. *Br J Cancer* **77**: 1838–1840

*© Cancer Research Campaign 1998*